# First Evaluation of Temporal and Spatial Fractionation in Proton Minibeam Radiation Therapy of Glioma-Bearing Rats

**DOI:** 10.3390/cancers13194865

**Published:** 2021-09-28

**Authors:** Annaïg Bertho, Ramon Ortiz, Marjorie Juchaux, Cristèle Gilbert, Charlotte Lamirault, Frederic Pouzoulet, Laura Polledo, Alethea Liens, Nils Warfving, Catherine Sebrie, Laurène Jourdain, Annalisa Patriarca, Ludovic de Marzi, Yolanda Prezado

**Affiliations:** 1 Institut Curie, Université PSL, CNRS UMR3347, Inserm U1021, Signalisation Radiobiologie et Cancer, 91400 Orsay, France; annaig.bertho@curie.fr (A.B.); ramon.ortiz@curie.fr (R.O.); marjorie.juchaux@curie.fr (M.J.); cristele.gilbert@curie.fr (C.G.); 2 Université Paris-Saclay, CNRS UMR3347, Inserm U1021, Signalisation Radiobiologie et Cancer, 91400 Orsay, France; 3 Translational Research Department, Institut Curie, Experimental Radiotherapy Platform, Université Paris Saclay, 91400 Orsay, France; charlotte.lamirault@curie.fr (C.L.); Frederic.pouzoulet@curie.fr (F.P.); 4 AnaPath GmbH, AnaPath Services, Hammerstrasse 49, 4410 Liestal, Switzerland; lpolledo@anapath.ch (L.P.); aliens@anapath.ch (A.L.); nwarfving@anapath.ch (N.W.); 5 CEA, CNRS, Inserm, Service Hospitalier Frédéric Joliot, BIOMAPS Université Paris-Saclay, 91401 Orsay, France; catherine.sebrie@universite-paris-saclay.fr (C.S.); laurene.jourdain@universite-paris-saclay.fr (L.J.); 6 Centre de Protonthérapie d’Orsay, Radiation Oncology Department, Campus Universitaire, Institut Curie, PSL Research University, 91898 Orsay, France; annalisa.patriarca@curie.fr (A.P.); ludovic.demarzi@curie.fr (L.d.M.); 7 Institut Curie, Campus Universitaire, PSL Research University, University Paris Saclay, INSERM LITO, 91898 Orsay, France

**Keywords:** proton minibeam radiation therapy, temporal fractionation, high-grade gliomas

## Abstract

**Simple Summary:**

Proton minibeam radiation therapy (pMBRT) is a novel therapeutic approach based on a distinct dose delivery method: the dose distributions follow a pattern with regions of peaks (high doses) and valleys (low doses). pMBRT was shown to be able to widen the therapeutic window in glioma-bearing rats. In previous studies the irradiation was performed in one single fraction. The work reported in this manuscript is the first evaluation detailing the response of glioma-bearing rats to a temporal fractionation in proton minibeam radiation therapy, delivered under a crossfire geometry. A significant increase of the median survival time was obtained when the dose was delivered over two sessions as opposed to in a single fraction. This result could facilitate the path towards pMBRT treatments.

**Abstract:**

(1) Background: Proton minibeam radiation therapy (pMBRT) is a new radiotherapy technique using spatially modulated narrow proton beams. pMBRT results in a significantly reduced local tissue toxicity while maintaining or even increasing the tumor control efficacy as compared to conventional radiotherapy in small animal experiments. In all the experiments performed up to date in tumor bearing animals, the dose was delivered in one single fraction. This is the first assessment on the impact of a temporal fractionation scheme on the response of glioma-bearing animals to pMBRT. (2) Methods: glioma-bearing rats were irradiated with pMBRT using a crossfire geometry. The response of the irradiated animals in one and two fractions was compared. An additional group of animals was also treated with conventional broad beam irradiations. (3) Results: pMBRT delivered in two fractions at the biological equivalent dose corresponding to one fraction resulted in the highest median survival time, with 80% long-term survivors free of tumors. No increase in local toxicity was noted in this group with respect to the other pMBRT irradiated groups. Conventional broad beam irradiations resulted in the most severe local toxicity. (4) Conclusion: Temporal fractionation increases the therapeutic index in pMBRT and could ease the path towards clinical trials.

## 1. Introduction

Proton minibeam radiation therapy (pMBRT) is a novel therapeutic approach [1] based on the use of narrow beamlets (0.5 to 1 mm) combined with a strong spatial modulation of the dose. In pMBRT, the dose profiles feature alternating regions of high doses (peaks) and low doses (valleys) [2,3,4]. The use of protons brings with it additional advantages: (i) a negligible dose after the Bragg peak (end of the proton path) is obtained, which helps in the preservation of healthy surrounding tissue and (ii) there is a possibility of delivering a homogeneous dose distribution within the tumor, while preserving the distribution of peaks and valleys in normal tissues. pMBRT has already shown a remar-kable preservation of normal brain tissues [5,6,7,8], including the sparing of cognitive, emotional, and motor processes [7]. Furthermore, pMBRT provides an equivalent or superior mean lifespan in glioma-bearing rats when compared to standard proton therapy (PT) [9,10,11]. All the evaluations performed thus far have delivered the dose in one sole fraction, using a unique (unilateral) array.

Standard radiation therapy (RT) usually employs temporal fractionation of the dose to profit from both a reoxygenation of the tumor and a redistribution of cells to more sensitive phases of the cell cycle (late G2 and M phase) [12]. Local tissue toxicity is also reduced due to the resulting increased time available for the repair of sublethal damage, in addition to the repopulation of cells fractions [12]. Thus, temporal fractionation could also help with and increase the inherent benefit of the spatial fractionation of the dose for normal tissue preservation. 

This manuscript highlights the effects that a temporal fractionation of pMBRT has on glioma-bearing rats. The main goal of this work was to investigate whether pMBRT could profit from the same advantages as observed in standard RT, whereby the dose is fractionated over time. In order to overcome the difficulties linked with the repositioning of the animals to a high (micrometric) mechanical precision as required in pMBRT, a crossfire geometry was used: two orthogonal arrays delivered over either one or two different days following the method described by Serduc et al. [13]. Therefore, this was also the first investigation of pMBRT irradiations performed from two different entry ports. The employment of several arrays would lead to a reduced dose to normal tissues and thereby enable the use of lower peak doses. 

## 2. Materials and Methods

Ethics statement: All animal experiments were conducted in accordance with the animal welfare and ethical guidelines of our institution. They were approved by French Ministry of Research (permit no 2019122418442057).

### 2.1. Tumor Inoculation 

The RG2-[D74] (ATCC® CRL-2433™) glioma cell line transfected with the luciferase gene was used. A number of 5000 RG2-Luc cells were suspended in 5µL DMEM and then injected intracranially into 6-weeks old Male Fischer 344 rats (Janvier Labs) using a Hamilton syringe through a burr hole in the right caudate nucleus (2.5 mm anterior to the ear bars, i.e., at the bregma site, 4.7 mm lateral to the midline and at a depth of 5.5 mm from the skull). Bioluminescence imaging (BLI) with an IVIS spectrum (Perker Elmer, Houten, The Netherlands) was performed to confirm the tumor’s presence before irradiation. For the BLI procedure, the rats were injected intraperitoneally with a concentration of 150 mg/kg (P/N 122799) of D-luciferin (Perkin Elmer) in 500 µL. The peak of luminescence was reached 25 min after injection. The presence of a tumor was confirmed when the bioluminescent signal overcame the background level. Thus, only the rats expressing a BLI signal significantly higher than that of the background on the day of the irradiation were included in the study. Based on the BLI signal, the rats were rando-mized into groups, assuring that each group had a similar BLI average signal. In this experiment, the tumor size was not directly measured by means of magnetic resonance imaging (MRI). The tumor sizes assessed in previous experiments by means of MRI, at similar levels of average BLI signal and days after inoculation, suggest that the tumors in this experiment were irradiated with at least 4–5 hot and cold spots. The lack of MRI imaging of each animal just before irradiation is one of the main limitations of this study.

### 2.2. Irradiations and Dosimetry 

The irradiations were carried out using the pencil beam scanning delivery mode at the Orsay proton therapy center [14]. A proton beam of 100 MeV was used, and optimized collimators for proton minibeam generations were employed [15]. A 6.5 cm-thick brass multislit collimator with 5 slits with a width of 400 ± 50 µm and a center-to-center distance (c-t-c) of 2800 µm was used.

**Figure 1:**
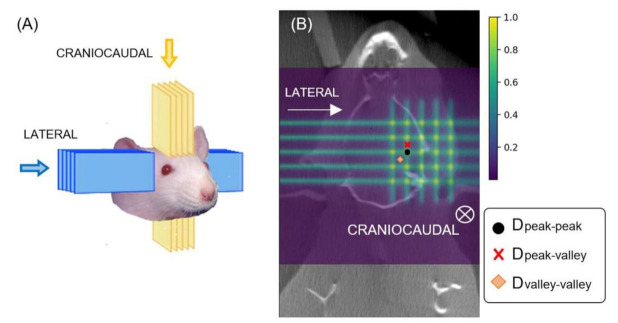
(**A**): pMBRT crossfire irradiation geometry. In blue, the lateral path of the beams. In yellow, the craniocaudal irradiation. (**B**) Coronal 2D dose maps in the crossfire pMBRT irradiations.

In the pMBRT irradiations, a crossfire geometry [16] was considered in order to avoid the blurring of the characteristic “peak and valley pattern” of pMBRT when the dose was to be delivered over two days. Two orthogonal arrays of minibeams intersecting at the target were considered: one in the craniocaudal direction and the other in the lateral direction, with skin-collimator distances of 7 cm and 6.5 cm, respectively. Figure 1 presents a sketch of the crossfire irradiation geometry.

With regards to the dosimetry evaluations, Monte Carlo simulations were carried out using with the TOPAS toolkit (v3.5 based on Geant4.10.7) [17]. Our Monte Carlo simulations had been previously benchmarked against experimental data [15]. The dose distributions were calculated using high resolution computer tomography images of a rat head of the same age of those to be irradiated. Figure 1 shows a 2D dose map in a coronal plane. Hereafter, D_peak-peak_ will refer to the hot spots where the two arrays cross, D_peak-valley_ to the areas corresponding to the peak of one of the arrays and the valley of the other, and D_valley-valley_ to the area in the center of the crossing region, which receives the minimal dose. Depth–dose curves can be found in the Supplemental material. See Figure S1. The Monte Carlo simulations were previously calibrated in terms of monitor units by means of experimental data measured with Gafchromic films and a microdiamond detector in water phantoms. These detectors had demonstrated good agreement in pMBRT, as highlighted in a previous work [3]. Since the irradiations were to be performed in the plateau region, where the average doses do not significantly vary in the first centimeters of depth, for the sake of simplicity in the Monte Carlo calibration, the dose prescription was performed at 1 cm-depth. Prior to the experiments, some film dosimetry experimental campaigns were carried out as a cross check to verify the irradiation conditions. Moreover, Gafchromic films were placed on the rats’ skin for quality assurance of the irradiation.

Five groups of animals were considered: 1. A non-irradiated tumor-bearing control group (*N* = 14); 2. A group that received crossfire irradiations in one unique fraction, with each of the arrays depositing 15 Gy average dose (*N* = 8); 3. A group using the same geometry and dose deposited as described in *2* but delivered over two different sessions spaced by 48 h. On day 1 a unilateral irradiation was delivered and 48 h later the animals received the craniocaudal irradiation (*N* = 7); 4. This group was the same as 3 but with a biological equivalent dose (BED) equal to one fraction in each of the arrays (20 Gy) (*N* = 6). The BED was calculated using Equation (1), where n refers to the number of fractions, d to the dose per fraction, and the ratio α/β is a measure of the fractionation sensitivity of the cells. A value of α/β of 10 was used as recommended in the literature for gliomas [18]. The average dose in the target was used to calculate BED; 5. This group received a conventional broad beam unilateral irradiation (30 Gy), *N* = 8. It should be noted that this corresponds to the same BED (120 Gy) as 20 Gy delivered in two fractions in BB.

(1) $$\mathrm{BED}=\mathrm{nd}(1+\frac{d}{\frac{\alpha }{\beta }})$$

Table 1 summarizes the groups and doses considered. A schematic representation of peaks, valleys, and average doses for the different groups can be found in the Supplemental material (Figure S2). The average dose in pMBRT is defined as the mean dose between the first and the last peaks. Groups 2, 4, and 5 have the same BED (120 Gy). See Table 1. The prescription of group 2 corresponds to the common practice in preclinical work of using the same physical dose independently of the temporal fractionation.

### 2.3. Animal Follow-Up

An anatomical MRI study was performed in the following groups: (i) number 2 (172 days after irradiation): (ii) number 4 (19, 33, 103, and 159 days after irradiation); and number 5 (6, 14, 21, 28, and 176 days after irradiation).

For each imaging session, a catheter was inserted into the tail vein for contrast agent administration. A 7-Tesla preclinical magnet (Bruker Avance Horizontal 7-T Bruker, Inc., Billerica, MA, United States) equipped with a 35 mm-diameter “bird-cage” antenna was employed. The employed sequences are described in our previous work [19].

To evaluate long-term effects, the animals were studied for 6 months. The clinical status of the animals was checked five times per week. Any rat showing classical adverse neurological signs related to tumor growth in the brain (i.e., substantial weight loss (>10% of weight within 24 h)) was humanely euthanized (with doletal injection or CO_2_ asphyxia). During rat necropsy, the brains were removed and fixed in 10% neutral-buffered formalin. After fixation, the brain tissues were embedded in paraffin wax and microtome sectioned at a thickness of 4 µm through the tumoral area before being stained with hematoxylin-eosin (HE) for histopathological evaluation. Microscopic evaluation was performed directly at the tumoral area and at the extratumoral region surrounding brain tissue in order to detect brain damage distal to the tumor. Histological changes were described according to distribution, severity, and morphologic character. Severity scores were assigned grade 1 to 5, as described in Appendix A.

All samples were image scanned by an Olympus Slideview VS200 slide scanner (Olympus, Tokyo, Japan) using a VS-264C camera (iDS, Obersulm, Germany) and 20× objective. Quantitative evaluation to determine the area of the tumor at the maximum diameter was performed using Olympus imaging and image analysis software cellSens v1.18 (Olympus, Tokyo, Japan). The histopathological (double-blinded) evaluation was carried out by board certified pathologists (European College of Veterinary Pathologists (ECVP)).

The statistical analysis of the histological findings was performed using the Brown–Forsythe one-way ANOVA test (Graphad Prism software, Graphad Software, San Diego, CA, USA).

## 3. Results

### 3.1. Clinical Symptoms

All the irradiated groups gained weight as a function of time. The irradiated animals of group 4 underwent a slight weight loss (10–15 gr) eight days after irradiation. Weight recovery started three days thereafter.

The comparative analysis of the weight evolution among the irradiated groups did not reveal any statistically significant differences (according to the ANOVA test) between the different irradiation configurations.

**Figure 2:**
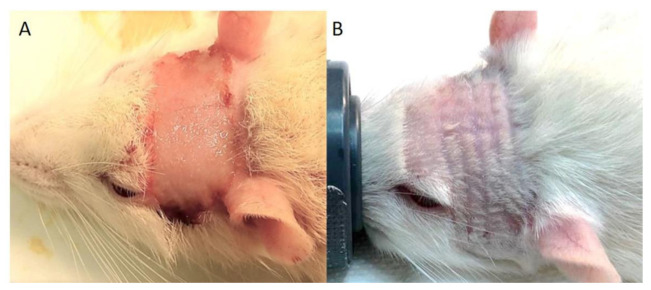
Radiation-induced skin toxicities after irradiation. (**A**) Rats receiving 30 Gy conventional broad beam irradiation developed severe cutaneous ulceration (radiation dermatitis) 11 to 12 days after irradiation. (**B**) Rats receiving crossfire pMBRT (in one or two fractions, 15 Gy or 20 Gy per array) did not show macroscopic signs of skin ulceration/radiation dermatitis. Nevertheless, they exhibited transient radiation-induced alopecia in the path of the beams, starting between 11 and 18 days after the last fraction.

None of the animals in any of the pMBRT groups exhibited macroscopic skin radiotoxicities. This is in contrast to the animals that received broad beam irradiations, who exhibited a severe cutaneous ulceration (radiation dermatitis) 11–12 days after irradiation, as depicted in Figure 2. pMBRT groups revealed only multifocal radiation-induced alopecia along the paths of the minibeams. A summary of the clinical symptoms of the animals in the groups is presented Table 2.

### 3.2. Survival Curves

**Figure 3:**
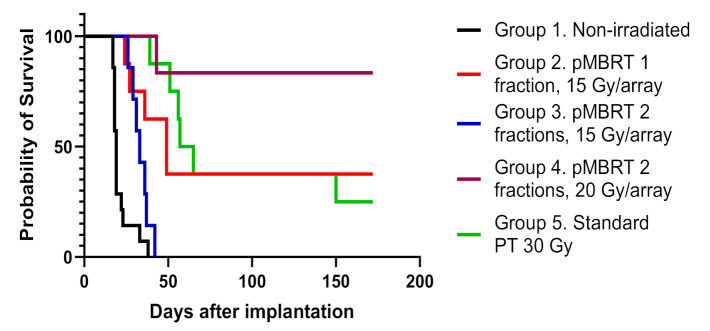
Comparison of the survival curves of the non-irradiated control (black line), standard proton therapy (green line, group 5), pMBRT delivered in one fraction (red line, group 2) or in two fractions (blue line, group 3) both with an average dose of 15 Gy/array, and pMBRT delivered in two fractions of 20 Gy/array (brown line, group 4). The differences were significant for the log-rank (Mantel–Cox) test (Chi square = 52.42, df = 4, *p* < 0.0001).

Figure 3 depicts the survival curves in which it can be seen that the survival of all irradiated groups was statistically significantly different from that of the control group (*p* ≤ 0.05). Table 3 reports the differences among the groups.

Interestingly, the irradiation of group 2, which received only one fraction of pMBRT, and group 5, which was conventionally irradiated with a standard broad beam, yielded statistically equivalent survival curves (according to the Log-rank (Mantel–Cox) test with *p* = 0.78), despite the differing dose distributions between the two groups. All irradiated groups, except group 3 (15 Gy/array in two fractions), exhibited a considerable proportion of long-term survivors, with the most promising results corresponding to group 2 (38%) and group 4 (83%). The latter probability of survival is the one of the best results ever obtained using exclusively radiation therapy to treat glioma-bearing rodents.

### 3.3. MRI Follow-Up 

In group 2 (pMBRT delivered in one fraction at 15 Gy/array), no tumor was observed in the MRI images 172 days after irradiation. However, one of the aforementioned images displayed a small area of T2 hyperintensity in the tumor implantation area. This image can be observed in Figure S3 of the supplemental material.

**Figure 4:**
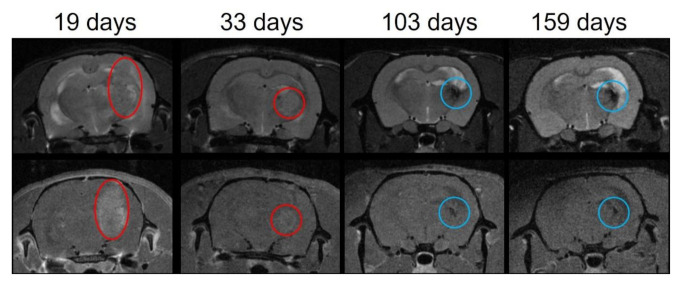
Example of the longitudinal MRI follow-up of one of the long-term survivors of group 4. Upper row: T2 images. Lower row: T1 RARE images after Gadolinium (Gd) injection. The tumor is identified by the red circle. A large tumor is observed at day 19 after irradiation (V = 104.82 mm^3^ on the T1 after Gd injection). The tumor size has diminished at day 33 after irradiation (V = 21.55 mm^3^ on the T1 after Gd injection). No tumor was observed at 103 nor 159 days after irradiation. The blue circle identified the lesion at the tumor bed area.

In group 4 (pMBRT, delivered in two fractions, at 20 Gy/array), no tumor was detected in any of the long-term survivors 159 days after irradiation. In 2 out of the 5 long-term survivors, there was some darkening, which is indicative of lesions (blue circles in Figure 4) in the ancient area of the tumor bed, compatible with the histopathological observations. See Section 3.4. Figure 4 shows the longitudinal MRI follow-up for one of those animals.

Long-term survivors in group 5 (standard broad beam) showed no tumor residue or radiation-induced lesions on MRI images at the end of the study.

### 3.4. Histopathological Evaluation 

The control group and most of the treated animals that were prematurely euthanized due to tumor growth exhibited a focal glioma, observed at level 3 of the Bolon scheme [20], located unilateral and close to the lateral ventricle either at the hippocampal area, dorsolateral thalamus, or parietal cortex.

**Figure 5:**
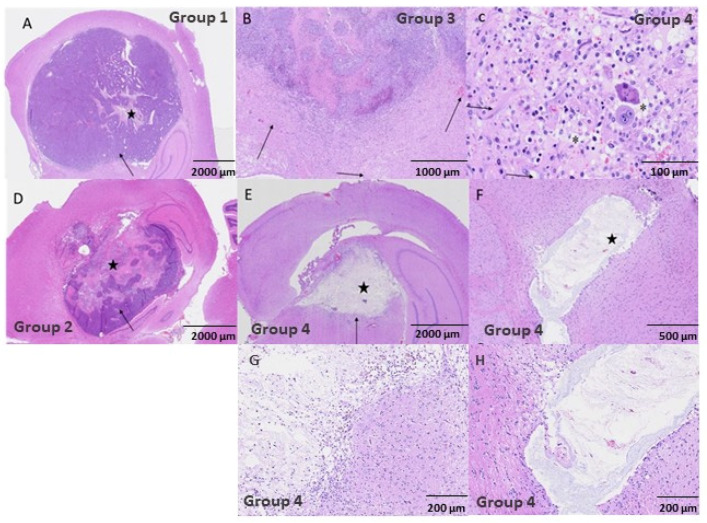
HE stained histopathology images. (**A**) corresponds to the non-irradiated control group with a scale bar of 2000 µm. There is a large glioma tumor (arrow) with a small necrotic area of necrosis and inflammation in the center (star). (**B**–**D**) depict the group 3 animals with scale bars of 1000 µm and 100 µm, respectively. In (**B**) there is marked inflammation around the tumor, with mild edema and hemorrhage indicated by the arrows. In (**C**) we observe peritumoral mixed inflammation with macrophages, lymphocytes, plasma cells, and neutrophils. The neuroparenchyma was expanded by edema and sites of spongiosis are depicted by the asterisks. Glial cells were reactive (arrows). The group 2 animals are shown in (**D**) with a scale bar of 2000 µm. There are glioma tumoral cells (arrow) with a large central area of necrosis (star). (**E**,**F**) are for group 4 animals with scales of 2000 µm and 5000 µm, respectively. In (**E**) we observe a central pseudo-cystic area of old necrosis (star) surrounded by inflammation (arrow). Panel F depicts a center of necrosis with only pale eosinophilic substance and necrotic cell debris (star), with minimal inflammatory peripheral infiltrate. (**G**,**H**) correspond to amplified images of the areas of interest in images (**E**,**F**), respectively.

The tumor area was on average larger in animals belonging to the control group and group 3 (15 Gy/array pMBRT delivered in two fractions). The animals from both of these groups died at early time points, as depicted in Figure S4 of the Supplemental material. Treatment with 20 Gy/array pMBRT over 2 days (group 4) resulted in 5 out of the 6 animals displaying a complete absence of tumoral cells at the end of the study period (total tumoral clearance), which corresponds to the longest average survival period after tumor implantation of all the groups evaluated in this study. At the area of the tumor implantation, only remnants of the resolved lesions were observed: Focal areas showed a pseudocystic-like appearance, characterized by central areas of necrosis from the tumor with only pale, eosinophilic substances and scarce necrotic cell debris of approximately 1 mm^2^, surrounded by inflammatory cells (in different degrees of severity). See Figure 5. The histologically intratumoral (IT) criterion were scored within the remnants of the resolved lesion.

**Figure 6:**
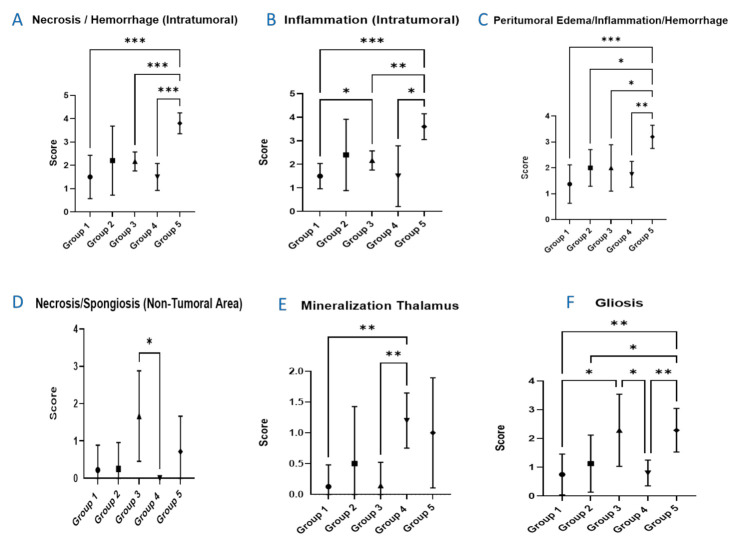
Presentation of the summary results from the histopathology evaluation. (**A**) intratumoral necrosis and hemorrhage. (**B**) intratumoral inflammation. (**C**) peritumoral edema, hemorrhage, and inflammation. (**D**) non-tumoral area of necrosis and spongiosis. (**E**) mineralization of the thalamus. (**F**) gliosis. Statistically significant differences (*p* < 0.05) between different groups are represented in the figure by asterisks. Group 1 represents the non-irradiated controls; Group 2 integrates the animals that received pMBRT in one fraction of 15 Gy/array; Group 3 includes the animals that received pMBRT in two fractions of 15 Gy/array; Group 4 depicts the animals that received pMBRT in two fractions of 20 Gy/array; and Group 5 includes the animals having received broad beam irradiations. Statistically significant differences are indicated by *, ** and *** for *p* values less or equal than 0.05, 0.01, and 0.001, respectively.

Figure 6 shows the quantification of the different histology findings as a function of the group. Within the tumors, there were eosinophilic areas with loss of cellular detail and accumulation of cellular debris (intratumoral necrosis, see Figure 5) and infiltrates of varying numbers of mixed inflammatory cells with abundant granulocytes, lymphocytes, and macrophages (inflammation, intratumoral). Peritumoral edema and hemorrhage were observed in different degrees of severity, and mixed cell inflammatory infiltrates were present around the tumors, consisting of granulocytes, lymphocytes, macrophages, and plasma cells (inflammation). Gliosis and often endothelial hypertrophy of varying severity were also noted in these areas. The greatest intratumoral necrosis and inflammation was observed in group 5 (standard broad beam). Furthermore, this group was also the most severely affected by the abovementioned peritumoral lesions (group 5 presented significantly higher intratumoral necrosis and hemorrhage, in addition to peritumoral edema/inflammation/necrosis as depicted in Figure 6). The minor presence of necrosis/spongiosis in non-tumoral areas was comparable in severity among all the groups, including the controls. This, however, was not the case for group 4. The presence of gliosis was observed more frequently in groups 3 and 5, in which the severity between the two groups was comparable and also significantly higher than that in group 4. Focal areas with deposits of mineralization of up to 50 µm (so-called cerebral calcification), were found within the thalamus in multiple animals, and were observed more frequently in the group 4 animals.

## 4. Discussion

Currently, technological advances in radiation delivery, including image guidance and particle therapy (i.e., proton therapy), have notably improved the tumor dose conformation, thus reducing the dose to the organs at risk [21,22]. However, the treatment of some radioresistant tumors, tumors close to a sensitive structure (e.g., central nervous system (CNS)), and pediatric cancers are still compromised due to the tolerances of normal tissues. The management of radioresistant brain tumors (i.e., glioblastoma multiforme (GBM)) is especially challenging due to the high morbidity of the CNS. Currently, the standard of care treatment for GBM patients is surgery followed by a combination of radiation and adjuvant chemotherapy with temozolomide (TMZ) [23]. The benefit of proton therapy for GBM was evaluated in a phase II clinical trial [24], in which a total equivalent dose of 90 Gy was delivered. An increased overall survival (20 months) was achieved at the price of high rates of clinically symptomatic neurotoxicity. A possible strategy to overcome normal tissue radiation-induced toxicity is to use distinct dose delivery methods. Accordingly, proton minibeam radiation therapy is an innovative approach, which has already proven its ability to remarkably minimize neurotoxicity [7,8]. pMBRT has already demonstrated a good tumor control effectiveness in glioma-bearing rats. Previously, all the studies on tumor control in pMBRT used one single fraction to avoid any blurring of the characteristic peak and valley pattern due to repositioning inaccuracies. However, temporal fractionation is usually employed in standard RT as it helps in tumor control by favoring tumor reoxygenation and cell cycle redistribution, while reducing normal tissue toxicities [25]. This manuscript reports on the first evaluation of the response of glioma-bearing rats to a temporal fractionation in pMBRT. In order to overcome the technical challenges (related to an overlap of beams) in achieving an accurate and robust re-irradiation with sub-millimetric precision, temporal fractionation was assessed by using a crossfire irradiation of two orthogonal arrays delivered over two different days following the method described by Serduc et al. [13].

The responses of glioma-bearing rats to crossfire irradiation delivered in one or two fractions were compared. A group irradiated with standard broad beam irradiations was also included. Our study shows that at the same average dose delivered in one fraction, crossfire pMBRT and proton broad beam irradiations led to statistically equivalent survival curves, despite the different dose target coverage in the two groups. This is coherent with the results of some previous studies [11]. However, pMBRT increases the therapeutic index since reduced neurotoxicity was reported when compared to that of the BB group (group 5). A significantly more severe induced neurotoxicity after standard broad beam irradiations was also observed in our previous evaluations compared to that of pMBRT [8].

Two fractions of crossfire pMBRT (group 3) with the same dose prescription as group 2 (delivered in one fraction) resulted in an increased survival compared to that the controls; however, it failed in obtaining total remission of the tumors, and no long-term survivors were obtained. The choice of using the same dose prescription as the irradiation performed in one fraction is a common practice in preclinical work. This could explain the lack of success of Serduc et al. in achieving long-term survivors [13].

Two fractions with 20 Gy/array/day (group 4) resulted in 83% long-term survivors with a total lack of tumoral cells, a factor 3.3 times higher than that with conventional broad beam irradiations. Despite the main limitations of this study, namely, low statistics in some groups and the lack of exact tumor volume measurements by MRI before irradiation (only BLI was available), this is one of the best results obtained thus far in glioma-bearing rats treated with pMBRT, even compared to some other spatially fractionated techniques, such as microbeam radiation therapy (MRT) [26]. One of the best results in MRT was also obtained using a crossfire geometry: Fernandez-Palomo also reported a complete remission in a mice melanoma model in 50% of the animals treated with crossfire X-rays MRT in three fractions [27]. In the evaluation of Fernandez-Palomo et al., the melanoma-bearing mice survived longer after irradiations with cross-fired microbeams in 3 fractions than they did after the unidirectional one-fraction irradiation. However, the different dose distributions used in the two temporal fractionation schemes do not allow for a clear conclusion to be drawn on its advantages. Regarding normal tissues, no significant excess of toxicity in group 4 with respect to the other groups was observed. Additionally, the toxicity was reduced with respect to standard broad beam irradiations. A higher mineralization of the thalamus was observed in this group compared to group 3. However, it should be noted that all animals in group 3 were sacrificed less than 2 months after irradiation due to tumor growth, thus corresponding to a shorter period of time in which to develop said mineralization.

Cerebral mineralization (calcification) is a well-known late radiation effect that often happens in the basal ganglia, more frequently at the thalamic area, especially in children [28,29]. Due to its focal nature, and the minimal-to-slight severity of this finding associated with the lack of brain damage, the clinical relevance is expected to be none or very minor at this point in time.

The only previous study evaluating temporally fractionated (4 fractions) pMBRT in normal tissues concluded that at the same level of integral dose, highly dose-modulated fractions spare more than low daily dose-modulated fractions [30], reflecting the advantages of a high precision repositioning between each fraction.

However, this study did not provide an assessment on the effects of temporally fractionating the dose in pMBRT as no comparison between one and several fractions was performed. Correspondingly, the use of crossfire geometries, such the one presented in this work, can help in overcoming the technical challenges of a high precision positioning, and make temporal fractionation more feasible in a potential future clinical context.

## 5. Conclusions

Temporal fractionation further improves the high therapeutic index of pMBRT in glioma-bearing animals. The number of long-term survivors when the dose was delivered in two fractions (83%) was 2.2 times higher than that when a single fraction of pMBRT was used. This factor was increased to 3.3 when compared to standard broad beam irradiations. Normal tissue damage remained minimal and was reduced with respect to standard irradiations. These results can facilitate the translation of pMBRT to patients’ treatments by enabling the splitting of the dose into several fractions and entry ports, using cross-fired arrays with lower peak doses than those used in the single fraction/single port studies.

## Figures and Tables

**Table 1:** Group distributions and doses.

Type of Irradiation	Group	Fractions	Dose Per Array (Gy)	Average Dose in the Target (Gy)	BED Target (Gy)	D_peak-peak_ (Gy)	D_peak-valley_ (Gy)	D_valley_valley_ (Gy)
Ctrl	1	0	0	0	0	-	-	-
pMBRT crossfire (2 arrays)	2	1	15 ± 1	30 ± 1	120 ± 6	59 ± 2	36 ± 1	10.6 ± 0.5
	3	2	15 ± 1	30 ± 1	75 ± 4	59 ± 2	36 ± 1	10.6 ± 0.5
	4	2	20 ± 1	40 ± 2	120 ± 6	79 ± 2	46 ± 1	14.2 ± 0.5
Broad beam (1 array)	5	1	30 ± 2	30 ± 2	120 ± 6	−	−	−

**Table 2:** Summary of clinical symptoms shown in the experimental groups.

Type of Irradiation	Dose (Gy) Per Array	Group	Transitory Weight Loss	Radiation Dermatitis	Radiation-Induced Alopecia
Ctrl	−	1	−	−	−
pMBRT crossfire	15 Gy	2 (1 fraction)	4/8 3 to 6 days	0/7	7/7
	15 Gy	3 (2 fractions)	7/7 3 to 5 days	0/8	8/8
	20 Gy	4 (2 fractions)	6/6 1 to 3 days	0/8	6/6
Broad beam (1 array)	30 Gy	5 (1 fraction)	4/8 2 to 4 days	8/8 11 to 12 days	−

**Table 3:** Differences in the survival curves of the different groups. “ns” refers to non-significant, one and three asterisks represent *p*-values less than 0.05 and 0.001, respectively.

Group	1	2	3	4	5
**1**		***	*	***	***
**2**	***		*	ns	ns
**3**	*	*		***	***
**4**	***	***	***		***
**5**	***	ns	*	ns	

***: statistically significant difference with *p* ≤ 0.001, *: statistically significant difference with *p* ≤ 0.05.

## Data Availability

The data presented in this study are available on request from the corresponding author.

## Supplementary Materials

The following are available online at https://www.mdpi.com/article/10.3390/cancers13194865/s1, Figure S1: Depth-dose curves, Figure S2: Schematic approximative representation of dose profiles per array at the tumor position for the different groups. Figure S3: Longitudinal MRI follow-up of one of the long-term survivals of group 2, Figure S4: Average tumor maximal area (µm^2^) and quantitative histomorphometry result for tumor size.

## Appendix A

Severity scores were assigned Grade 1 to 5.

Grade 1, Minimal	This corresponds to a histopathologic change ranging from inconspicuous to barely noticeable but so minor, small, or infrequent as to warrant no more than the least assignable grade. For multifocal or diffusely distributed lesions, this grade was used for processes where less than approximately 10% of the tissue in an average high-power field was involved.
Grade 2, Slight	This corresponds to a histopathologic change that is a noticeable but not a prominent feature of the tissue. For multifocal or diffusely distributed lesions, this grade was used for processes where between approximately 10% and 25% of the tissue in an average high-power field was involved.
Grade 3, Moderate	This corresponds to a histopathologic change that is a prominent but not a dominant feature of the tissue. For multifocal or diffusely distributed lesions, this grade was used for processes where between approximately 25% and 50% of the tissue in an average high-power field was involved.
Grade 4, Marked	This corresponds to a histopathologic change that is a dominant but not an overwhelming feature of the tissue. For multifocal or diffusely distributed lesions, this grade was used for processes where between approximately 50% and 95% of the tissue in an average high-power field was involved.
Grade 5, Severe	This corresponds to a histopathologic change that is an overwhelming feature of the tissue. For multifocal or diffusely distributed lesions, this grade was used for processes where greater than approximately 95% of the tissue in an average high-power field was involved.

## References

[1] Prezado Y., Fois G.R. (2013). Proton-minibeam radiation therapy: A proof of concept. Med. Phys..

[2] Peucelle C., Nauraye C., Patriarca A., Hierso E., Fournier-Bidoz N., Martinez-Rovira I., Prezado Y. (2015). Proton minibeam radiation therapy: Experimental dosimetry evaluation. Med. Phys..

[3] Guardiola C., De Marzi L., Prezado Y. (2020). Verification of a Monte Carlo dose calculation engine in proton minibeam radiotherapy in a passive scattering beamline for preclinical trials. Br. J. Radiol..

[4] Meyer J., Eley J., Schmid T.E., Combs S.E., Dendale R., Prezado Y. (2019). Spatially fractionated proton minibeams. Br. J. Radiol..

[5] Girst S., Greubel C., Reindl J., Siebenwirth C., Zlobinskaya O., Walsh D.W.M., Ilicic K., Aichler M., Walch A., Wilkens J.J. (2016). Proton minibeam radiation therapy reduces side effects in an in vivo mouse ear model. Int. J. Radiat. Oncol. Biol. Phys..

[6] Zlobinskaya O., Girst S., Greubel C., Hable V., Siebenwirth C., Walsh D.W.M., Multhoff G., Wilkens J.J., Schmid T.E., Dollinger G. (2013). Reduced side effects by proton microchannel radiotherapy: Study in a human skin model. Radiat. Environ. Biophys..

[7] Lamirault C., Doyere V., Juchaux M., Pouzoulet F., Labiod D., Dendale R., Patriarca A., Nauraye C., Le Dudal M., Jouvion G. (2020). Short and long-term evaluation of the impact of proton minibeam radiation therapy on motor, emotional and cognitive functions. Sci. Rep..

[8] Prezado Y., Jouvion G., Hardy D., Patriarca A., Nauraye C., Bergs J., Gonzalez W., Guardiola C., Juchaux M., Labiod D. (2017). Proton minibeam radiation therapy spares normal rat brain: Long-term clinical, radiological and histopathological, analysis. Sci. Rep..

[9] Prezado Y., Jouvion G., Patriarca A., Nauraye C., Guardiola C., Juchaux M., Lamirault C., Labiod D., Jourdain L., Sebrie C. (2018). Proton minibeam radiation therapy widens the therapeutic index for high-grade gliomas. Sci. Rep..

[10] Prezado Y., Jouvion G., Guardiola C., Gonzalez W., Juchaux M., Bergs J., Nauraye C., Labiod D., De Marzi L., Pouzoulet F. (2019). Tumor control in RG2 glioma-bearing rats: A comparison between proton minibeam therapy and standard proton therapy. Int. J. Radiat. Oncol. Biol. Phys..

[11] Lamirault C., Brisebard E., Patriarca A., Juchaux M., Crepin D., Labiod D., Pouzoulet F., Sebrie C., Jourdain L., Le Dudal M. (2020). Spatially modulated proton minibeams results in the same increase of lifespan as a uniform target dose coverage in F98-glioma-bearing rats. Radiat. Res..

[12] Steel G.G., McMillan T.J., Peacock J.H. (1989). The 5Rs of radiobiology. Int. J. Radiat. Biol..

[13] Serduc R., Brauer-Krisch E., Bouchet A., Renaud L., Brochard T., Bravin A., Laissue J.A., Le Duc G. (2009). First trial of spatial and temporal fractionations of the delivered dose using synchrotron microbeam radiation therapy. J. Synchrotron. Radiat..

[14] De Marzi L., Da Fonseca A., Moignier C., Patriarca A., Goudjil F., Mazal A., Buvat I., Hérault J. (2019). Experimental characterisation of a proton kernel model for pencil beam scanning techniques. Phys. Med..

[15] De Marzi L., Patriarca A., Nauraye C., Hierso E., Dendale R., Guardiola C., Prezado Y. (2018). Implementation of planar proton minibeam radiation therapy using a pencil beam scanning system: A proof of concept study. Med. Phys..

[16] Brauer-Krisch E., Requardt H., Regnard P., Corde S., Siegbahn E., LeDuc G., Brochard T., Blattmann H., Laissue J., Bravin A. (2005). New irradiation geometry for microbeam radiation therapy. Phys. Med. Biol..

[17] Perl J., Shin J., Schumann J., Faddegon B., Paganetti H. (2012). TOPAS: An innovative proton Monte Carlo platform for research and clinical applications. Med. Phys..

[18] Qi X.S., Schultz C.J., Li X.A. (2006). An estimation of radiobiologic parameters from clinical outcomes for radiation treatment planning of brain tumor. Int. J. Radiat. Oncol. Biol. Phys..

[19] Prezado Y., Dos Santos M., González W., Jouvion G., Guardiola C., Heinrich S., Labiod D., Juchaux M., Jourdain L., Sébrié C. (2017). Transfer of minibeam radiation therapy into a cost-effective equipment for radiobiological studies: A proof of concept. Sci. Rep..

[20] Bolon B., Garman R.H., Pardo I.D., Jensen K., Sills R.C., Roulois A., Radovsky A., Bradley A., Andrews-Jones L., Butt M. (2013). STP position paper: Recommended practices for sampling and processing the nervous system (brain, spinal cord, nerve, and eye) during nonclinical general toxicity studies. Toxicol. Pathol..

[21] Bernier J., Hall E.J., Giaccia A. (2004). Radiation oncology: A century of achievements. Nat. Rev. Cancer.

[22] Garibaldi C., Jereczek-Fossa B.A., Marvaso G., Dicuonzo S., Rojas D.P., Cattani F., Starzyńska A., Ciardo D., Surgo A., Leonardi M.C. (2017). Recent advances in radiation oncology. Ecancer Med. Sci..

[23] Stupp R., Mason W.P., van den Bent M.J., Weller M., Fisher B., Taphoorn M.J., Belanger K., Brandes A.A., Marosi C., Bogdahn U. (2005). Radiotherapy plus concomitant and adjuvant temozolomide for glioblastoma. N. Engl. J. Med..

[24] Fitzek M.M., Thornton A.F., Rabinov J.D., Lev M.H., Pardo F.S., Munzenrider J.E., Okunieff P., Bussière M., Braun I., Hochberg F.H. (1999). Accelerated fractionated proton/photon irradiation to 90 cobalt gray equivalent for glioblastoma multiforme: Results of a phase II prospective trial. J. Neurosurg..

[25] Shibamoto Y., Iwata H. (2020). The quest for optimal fractionation schedules in stereotactic radiotherapy. Cureus.

[26] Fernandez-Palomo C., Fazzari J., Trappetti V., Smyth L., Janka H., Laissue J., Djonov V. (2020). Animal models in microbeam radiation therapy: A scoping review. Cancers.

[27] Fernandez-Palomo C., Trappetti V., Potez M., Pellicioli P., Krisch M., Laissue J., Djonov V. (2020). Complete remission of mouse melanoma after temporally fractionated microbeam radiotherapy. Cancers.

[28] Abayomi O., Chun M.S., Kelly K. (1990). Cerebral calcification and learning disabilities following cranial irradiation for medulloblastoma. J. Natl. Med. Assoc..

[29] Carl U.M., Hartmann K.A. (2002). Heterotopic calcification as a late radiation effect: Report of 15 cases. Br. J. Radiol..

[30] Sammer M., Dombrowsky A.C., Schauer J., Oleksenko K., Bicher S., Schwarz B., Rudigkeit S., Matejka N., Reindl J., Bartzsch S. (2021). Normal tissue response of combined temporal and spatial fractionation in proton minibeam radiation therapy. Int. J. Radiat. Oncol Biol. Phys..

