# Supplementary material for: First Evaluation of Temporal and Spatial Fractionation in Proton Minibeam Radiation Therapy of Glioma-Bearing Rats
Source: Cancers (Basel). 2021 Sep 28;13(19):4865. doi: 10.3390/cancers13194865 (PMC8507607; doi:10.3390/cancers13194865)
Supplement: Supplementary file 1 [file cancers-13-04865-s001.zip › cancers-1386757-supplementary.pdf]

Supplementary Materials: First Evaluation of Temporal and Spatial Fractionation in Proton Minibeam Radiation Therapy of Glioma-Bearing Rats

Annaïg Bertho, Ramon Ortiz, Marjorie Juchaux, Cristèle Gilbert, Charlotte Lamirault, Frederic Pouzoulet, Laura Polledo, Alethea Liens, Nils Warfving, Catherine Sebrie, Laurène Jourdain, Annalisa Patriarca, Ludovic de Marzi and Yolanda Prezado

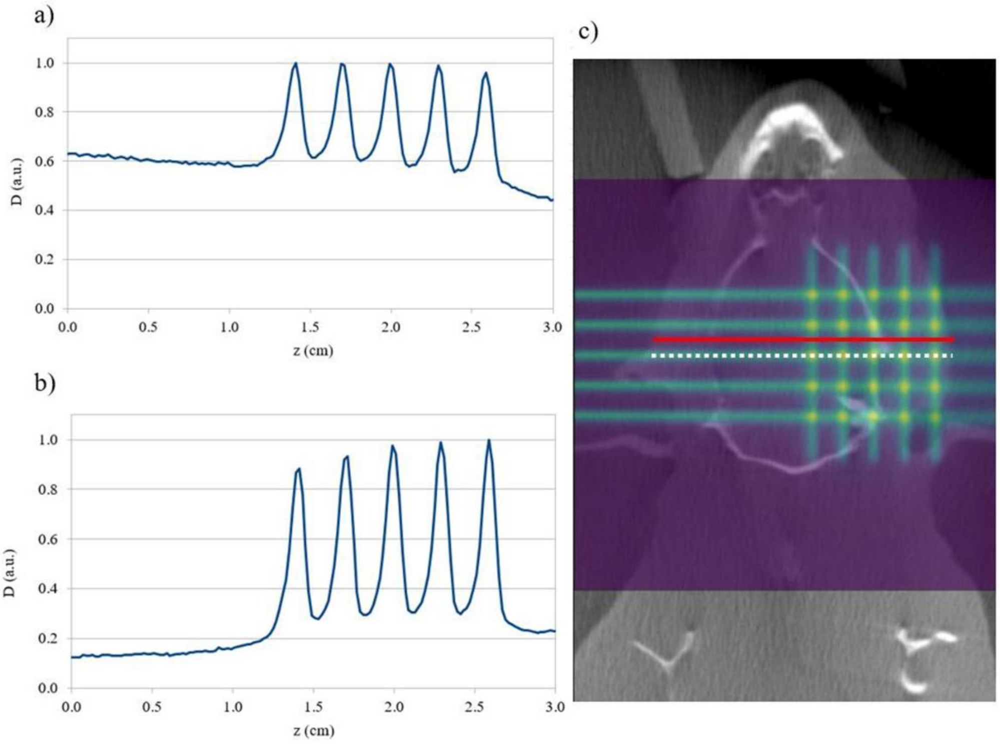

Figure S1. Depth-dose curves. (**a**) Dose profile of the craniocaudal array intersecting the central peak of the lateral array (white dotted line in (**c**)), and (**b**) dose profile of the craniocaudal array intersecting the central valley of the lateral array (red solid line in (**c**)).

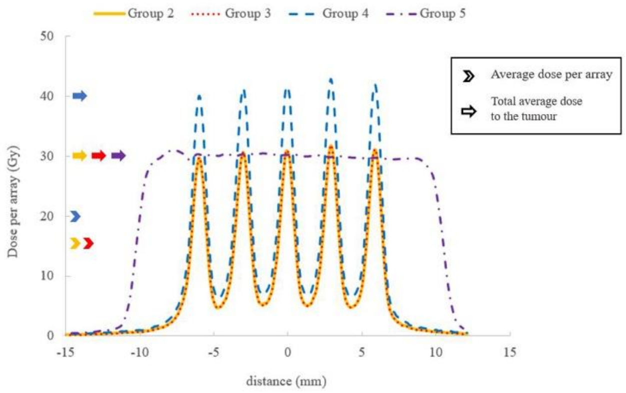

Figure S2. Schematic approximative representation of dose profiles per array at the tumor position for the different groups. The average doses of each group are represented by arrows.

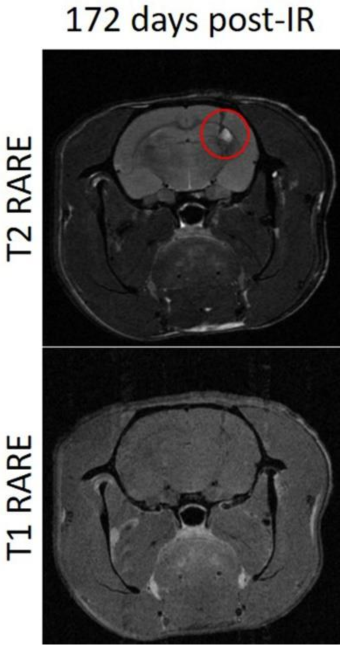

Figure S3. Longitudinal MRI follow up of one of the long-term survivals of group 2. Upper row: T2 images. Lower row: T1 RARE images after Gadolinium (Gd) injection. The red circle shows a small area of hyperintensity in T2 in the tumor implantation area.

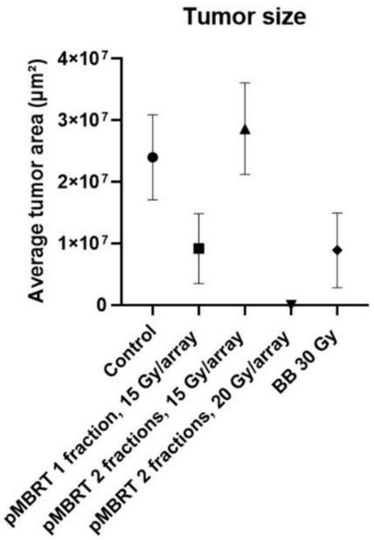

Figure S4. Average tumor maximal area (µm²), quantitative histomorphometry result for tumor size.
